# Identification, phylogenetic analysis and expression profile of an anionic insect defensin gene, with antibacterial activity, from bacterial-challenged cotton leafworm, *Spodoptera littoralis*

**DOI:** 10.1186/1471-2199-12-47

**Published:** 2011-11-09

**Authors:** AlaaEddeen M Seufi, Elsayed E Hafez, Fatma H Galal

**Affiliations:** 1 Department of Entomology, Faculty of Science, Cairo university, 9 Gamaa St. Giza, 12613, Egypt; 2 Department of Molecular plant pathology, ALCADRI, City for Scientific Research and Technology Application, New Borg ElArab, Alex, 21934, Egypt

## Abstract

**Background:**

Defensins are a well known family of cationic antibacterial peptides (AMPs) isolated from fungi, plants, insects, mussels, birds, and various mammals. They are predominantly active against gram (+) bacteria, and a few of them are also active against gram (-) bacteria and fungi. All insect defensins belonging to the invertebrate class have a consensus motif, C-X_5-16_-C-X_3_-C-X_9-10_-C-X_4-7_-CX_1_-C. Only seven AMPs have already been found in different lepidopteran species. No report was published on the isolation of defensin from the Egyptian cotton leafworm, *Spodoptera littoralis*.

**Results:**

An anionic defensin, termed *Spli*Def, was isolated from the haemolymph of the cotton leafworm, *S. littoralis*, after bacterial challenge using differential display technique. Based on sequence analyses of the data, specific primers for full length and mature peptide of defensin were designed and successfully amplified 471 and 150 bp amplicons. The integration of the results revealed that the 471 bp-PCR product has one open reading frame (*orf*) of 303 bp long, including both start codon (AUG) and stop codon (UGA). The deduced peptide consists of a 23-residues signal peptide, a 27-residues propeptide and a 50-residues mature peptide with the conserved six-cysteine motif of insect defensins. Both haemolymph and expressed protein exhibited antibacterial activities comparable to positive control. The RT-qPCR indicated that it was more than 41-folds up-regulated at 48 h p.i.

**Conclusion:**

Our results highlight an important immune role of the defensin gene in *Spodoptera littoralis *by cooperating with other AMPs to control bacterial infection.

## Background

The growing problem of resistance of microorganisms to current antibiotics has fostered the search for novel antimicrobial therapies [1]. Particularly interesting are antimicrobial peptides (AMPs) discovered as components of unspecific innate mechanisms of infection fighting in humans and animals [2]. AMPs play a crucial role in innate immune systems of invertebrates, which do not have an adaptive immunity. Insects protect themselves from pathogens and parasites via a powerful innate immune system. Insect innate immune responses include cellular and humoral responses, and humoral responses contain melanization and synthesis of antimicrobial peptides (AMPs). The insect immune responses are based on: the recognition of the pathogen as nonself, the induction of suitable genes and biochemical pathways that result in the production of a potent arsenal of low molecular weight AMPs [3,4]. Most of these peptides (AMPs) are produced in the fat body or haemocytes of the insect and released into the haemolymph. Insect AMPs are divided into three groups in accordance to their amino acid sequence and structural features: (i) cecropins which are linear peptides that form α-helix and lack cysteine residues; (ii) defensins which have a characteristic six to eight conserved cysteine residues that form a stabilizing array of three or four intramolecular disulfide bridges and three domains consisting in a flexible amino-terminal loop, a central α-helix and a carboxyl-terminal antiparallel β-sheet [5-7]; and (iii) peptides with an overrepresentation of proline and/or glycine residues, e.g., lebocins and moricins [7].

Defensins have been isolated from fungi, plants, insects, mussels, birds, and various mammals. They are predominantly active against gram (+) bacteria, and a few of them are also active against gram (-) bacteria and fungi. Regarding the spacing pattern of cysteines, defensins are divided into plant, invertebrate, α, β-, and -subfamilies [8]. All insect defensins belonging to the invertebrate class have a consensus motif, C-X_5-16_-C-X_3_-C-X_9-10_-C-X_4-7_-CX_1_-C. To date, hundreds of AMPs have been described in insects and a lot of different nucleotide and amino acid defensin or defensin-like sequences from many insect species were registered by NCBI data base. However, only seven authors reported the presence of defensin-like peptides in Lepidoptera [8-14]. Several AMPs have already been found in different species of *Spodoptera*. These include moricins, cecropins and defensin, but no report was published on the isolation of defensin from the Egyptian cotton leafworm, *Spodoptera littoralis*. Therefore, the main objective of the present research was to investigate the immune responses of the Egyptian *S. littoralis*, to bacterial challenge. Here we report the isolation, sequence characterization, phylogenetic analysis, antimicrobial activity and expression profile of a defensin gene from the haemolymph of *S. littoralis*.

## Results

### Differential display (DD-PCR) using primers corresponding to well known defense genes

**Figure 1:**
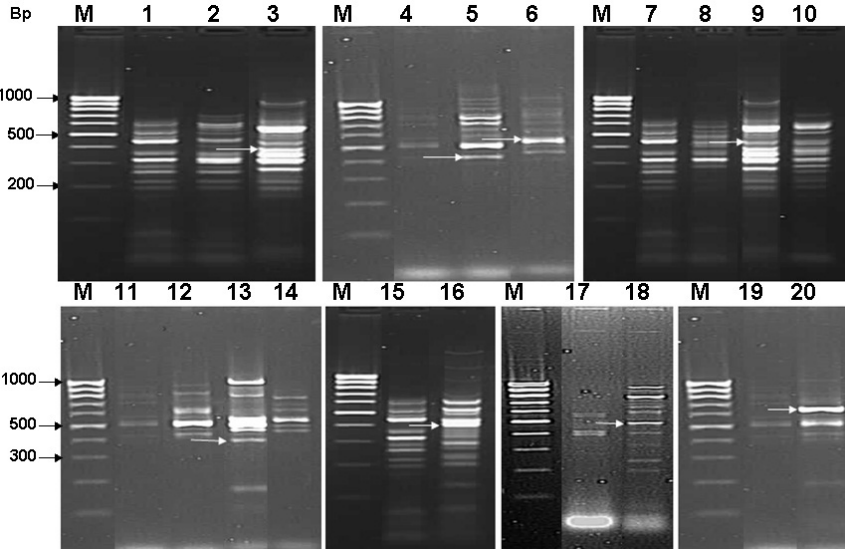
**Representative 1.5% agarose gels of DD-PCR patterns generated from control and *S. aureus*, *E. coli *and *S. sanguinis*-challenged haemolymph samples using 10 primers corresponding to well known defense genes**. Lane M: DNA marker 100 bp Ladder, lanes 1, 4, 7, 11, 15, 17 and 19: controls of different treatments, lanes 2, 3 and 5, 6: 24 and 48 h post-infection by *E. coli*, Lanes 8, 9, 10 and 12, 13, 14: 24, 48 and 72 h post-infection by *S. sanguinis *and Lanes 16, 18, 20: 42 h post-infection by *S. aureus*. Arrows refer to differentially displayed sequenced bands.

As the identification of the induced antibacterial genes was the main objective of this study, differential display technique was used to characterize the genetic variation (at RNA level) between bacterial-challenged and control *S. littoralis*. Figure 1 shows the results of differentially displayed cDNAs of bacterial-challenged and control insects using 10 primers (Table 1) corresponding to well known defense genes. Haemolymph samples were differentially displayed at 24, 48 and/or 72 h post-infection (p.i.) with *S. aureus, S. sanguinis *and *E. coli *bacterial strains. It was observed that *S. aureus*-challenged insects died 24 h p.i., *E. coli*-challenged insects died 48 h p.i. and *S. sanguinis*-challenged insects died 72 h p.i. Differential display results revealed that the average number of bands per sample was 4.1 bands. The total number of bands resolved in 1.5% agarose gel for both control and challenged insects was 332 (molecular size ranged from > 1000 to ~80 bp). 146 polymorphic bands (44%) were differentially displayed with 7 of the used primers. Eight reproducible, treatment-induced bands were cloned and sequenced using M_13 _universal primer.

**Table 1:** Key table for the primers used in DD-PCR study providing their names, origin and sequences.

Primer name	Origin	Sequence (5' -- 3')
CHIF	Chitinase-based	TGCCTTTGATTCAGTCATC

CHIR	Chitinase-based	AATAATCGACTCCAATACG

EGF	Endoglucanase-based	TCCGGGTATGTTATGGAAGA

EGR	Endoglucanase-based	GGCCATCCACTCTCAGACACA

LECF	Lectin-based	ATGGGATCCAAGCAACAGAG

LECR	Lectin-based	ATCCTTCAAAGACACAATGTCG

IDF	Insect defensin-based	CCAAATGCCTCGTCATCT

IDR	Insect defensin-based	ATTAGAGTCAAGCTAAAAGGG

HDF	Human defensin-based	TTATTTCTTTCTTCGGCAGC

HDR	Human defensin-based	GGAGCCCTTTCTGAATCCGCA

FLDefF	Full length defensin	GTTCGTCTATTTTTGTGCCG

FLDefR	Full length defensin	ACTTAAAAATCTATCATTGGCGTCA

MPDefF	Mature peptide defensin	GTTTCATGCGATTTCGAGGAAGCC

MPDefR	Mature peptide defensin	TCATGTGTAGGTATTTGTGTACC

PromF	Random primer	GGTCCCTGACTGATCCCTGG

PromR	Flanking region of cDNA	CTCATTATTTATATAACCTTAAC

All oligonucleotides were synthesized by Invitrogen, USA and HPLC purified.

### Primer design, RT-PCR amplification and cloning of defensin gene

**Figure 2:**
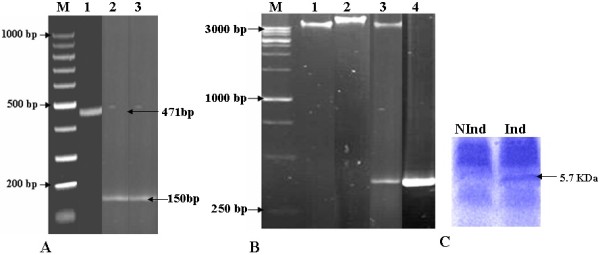
**Gel electrophoresis showing: Panel A: positive PCR representing full length and mature peptide (471 and 150 bp, respectively), Panel B: clone *PCR-Spli*Def after insert release with *EcoR*I, and PCR confirmation**. Lanes 1, 2, 3 and 4 show empty *PCR-TOPO, E. coli *harbouring *PCR-Spli*Def, *PCR-Spli*Def after digestion with *EcoR*I and positive control (471 bp amplified cDNA segment from *Spli*Def), respectively. PCR mix without DNA was used as negative control. Lanes M: DNA Markers. The size of the bands is shown in bp. Panel C: 12% Tricine SDS-PAGE showing the expressed protein after induction with IPTG.

Specific primers for the full length and mature peptide of defensin gene were designed. These primers would be used later in the following reactions during this study. Nucleotide sequence of the used primers was illustrated in Table 1. PCR was optimized for each primer set and primers successfully produced positive PCR amplicons of 471 and 150 bp for the full length and mature peptide sequences, respectively (Figure 2A). The full length fragment includes one open reading frame (*orf*) of defensin gene (positions 125 (AUG) - 427 (UGA)). Subsequently this segment (*Spli*Def) was cloned into *PCR-TOPO *vector (Figure 2B, lane 2) and transformed cells were tested with PCR using the same primers (Figure 2B, lane 4). Using this screening method, clone *PCR-TOPOSpli*Def was tested as positive (Figure 2B, lane 4). A PCR product corresponding to the mature defensin peptide was also cloned into *p*PROEXTM HTa expression vector and transformed DH_5α _cells were tested as positive (Figure 2A, lane 3).

### Nucleotide sequence and sequence analyses

**Figure 3:**
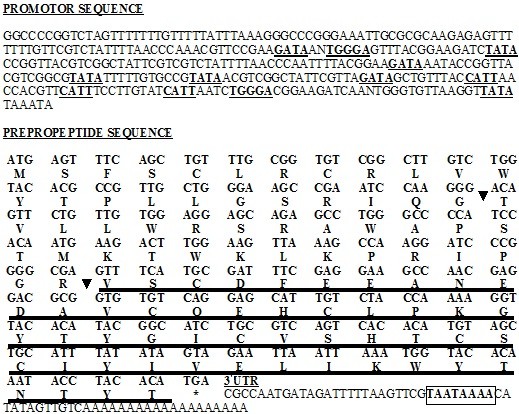
**Nucleotide and corresponding deduced amino acid sequence of *S. littoralis *defensin gene (*Spli*Def)**. In promoter sequence, TATA, CATT, IL-6-RE and GATA transcription factors are underlined. Cleavage sites of signal and propeptides are indicated by arrows. Mature peptide is double underlined. Asterisk indicates the stop codon. Boxed sequence represents the putative polyadenylation signal.

Nucleotide sequences of *Spli*Def and its deduced amino acid sequence are shown in Figure 3. A single *orf *that could encode a polypeptide of 100 amino acids was detected for *Spli*Def. One stop codon was found at the 3' end of the sequence. The flanking region of the initiation codon ATG is ATAATGAG, and the length of 3 untranslated region was 45 bp before the poly (A) track (Figure 3). TATA box as well as GATA, IL-6-RE, and CATT recognition sites were detected in the sequence of promoter region of defensin 321 bp upstream from the start codon. The putative polyadenylation sequence AATAAAA was located 25 bp downstream from the stop codon (Figure 3). The identified defensin *orf *includes signal peptide (69 bp), propeptide (81 bp) and mature peptide (150 bp). The deduced *Spli*Def polypeptide (prepropeptide) contains 14 strongly basic, 7 strongly acidic, 48 hydrophobic and 31 polar amino acids. The calculated molecular masses of the full length and mature defensins, were 11.6 and 5.7 KDa, respectively, and the calculated isoelectric points (PIs) were 8.5 and 4.3, respectively. The net charges at pH 7.0 were 4.8 and -5.1, respectively. The defensin prepropeptide was less stable (Instability Index (II): 33.32) than its mature peptide (II: 21.09). Ratios of hydrophilic residues were 31 and 30% for prepropeptide and mature defensin peptide, respectively.

**Figure 4:**
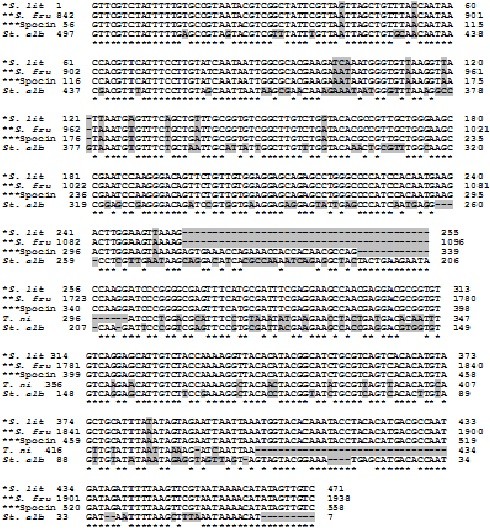
**Comparison of *defensin *nucleotide sequence from *S. littoralis *(Acc# **HQ603825**) with *S. frugiperda *defensin, spodoptericin, *T. ni *defensin and *St. albicosta *sequence (Acc# **AY128091, AY238439, EU016385** and **EZ596498**, respectively)**. *S. lit: *S. littoralis *defensin, **S. fru: *S. frugiperda *defensin and ***Spocin: *S. frugiperda *spodoptericin. Gaps and different nucleotides are shaded.

On comparing *Spli*Def nucleotide sequence (Acc# HQ603825) to *S. frugiperda *defensin and spodoptericin, *T. ni *defensin and *St. albicosta *sequence (Acc# AY128091, AY238439, EU016385 and EZ596498, respectively), 14, 14, 36 and 112 different nucleotides; zero, zero, one and 9 gaps; and 29, 40, zero and zero additions were observed throughout the compared DNA segments (Figure 4).

In addition, the deduced amino acid sequence of *Spli*Def was blasted to all defensin-related sequences in GenBank database. Blast search created significant alignment with 13 insect-published peptide sequences (9 defensins, 2 spodoptericins and other 2 mRNA products). The *Spli*Def putative peptide exhibited 96% identity with *S. frugiperda *defensin and 2 spodoptericins (Acc# AAM96925, AAQ18894 and AAQ18895, respectively), 67% identity with 3 *Bombyx mori *defensin-like proteins (Acc# NP_001037370, AAZ38358 and BAG48202), 75, 66, 40, 40 and 48% identity with the defensins of *T. ni*, *Manduca sexta*, *Lutzomyia longipalpis *and *Anopheles *sp. (Acc# ABV688523, ACX49766, ABR28349, ABV60344, respectively), and 31% identity with both *Drosophila yakuba *mRNA products (Acc# XP_002100630 and EDX01738).

**Figure 5:**
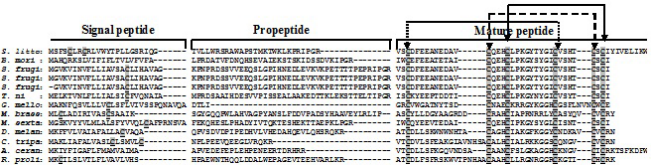
**Alignment of *Spli*Def (HQ603825) deduced amino acid sequence with other insect defensins**. The conserved cysteine residues are shaded. AAZ38358: *B. mori*, AAM96925: *S. frugiperda*, AAQ18895: *S. frugiperda *spodoptericin, AAQ18894: *S. frugiperda *spodoptericin, ABV68852: *T. ni*, AAS19170: *G. mellonella*, AAL69980: *Mamestra brassicae*, ACX49766: *Manduca sexta*, CAA81760: *Drosophila melanogaster*, ABP97087: *Copris tripartitus*, ACH96412: *Apis cerana cerana*, AAO74624: *Rhodnius prolixus*.

On comparing amino acid sequence of the putative polypeptide of *Spli*Def (HQ603825) to its corresponding sequences of *B. mori*, *S. frugiperda*, *S. frugiperda *spodoptericin, *S. frugiperda *spodoptericin, *T. ni*, *G. mellonella*, *Mamestra brassicae*, *M. sexta*, *D. melanogaster*, *Copris tripartitus*, *Apis cerana cerana *and *Rhodnius prolixus *(Acc# AAZ38358, AAM96925, AAQ18895, AAQ18894, ABV68852, AAS19170, AAL69980, ACX49766, CAA81760, ABP97087, ACH96412 and AAO74624, respectively), 6 conserved cysteine residues were observed throughout the 13 compared putative polypeptides (Figure 5). It is noteworthy to mention that *Spli*Def amino acid sequence contains 8 cysteine residues (2 residues in the signal peptide sequence and other 6 residues in the mature peptide). These are comparable to *M. brassicae *and *C. tripartitus *conserved cysteine motifs (Figure 5). *B. mori *and *A. cerana *defensins were observed to have only 6 conserved cysteine motifs. Meanwhile, all other compared defensins contains more than 6 (7 or 8) conserved cysteine motifs (Figure 5). The spacing pattern of *Spli*Def six-cysteine residues motif was C-X_10_-C-X_3_-C-X_9_-C-X_4_-C-X_1_-C, which was consistent with the consensus motif of invertebrate defensins. In addition to the precise conservation of the six cysteines, at least six residues within the cysteine motif of *Spli*Def, His^77^, Leu^79^, Lys^81^, Gly^82^, Tyr^83 ^and Gly^86 ^were found to be conserved at equivalent positions in the sequences of some other insect defensins (Figure 5).

Primary, secondary structure analyses, post-translational modifications and topology predictions revealed that amino acid sequence of the putative *Spli*Def peptide has 3 signal peptide cleavage sites (between positions 23-24/32-33/34-35), three O-GlcNAcylated residues (1 Ser and 2 Thr at positions 80, 37 and 100, respectively) and one potential glycated lysine at position 39. Two phosphorylation sites (Ser: 1 at position 52, Thr: 1 at position 40) and 13 (7 S, 2 Y and 4 T) kinase specific phosphorylation sites (highest score: 0.89 PKC at position 40) were predicted.

### Phylogenetic analyses of the *Spli*Def sequence

**Figure 6:**
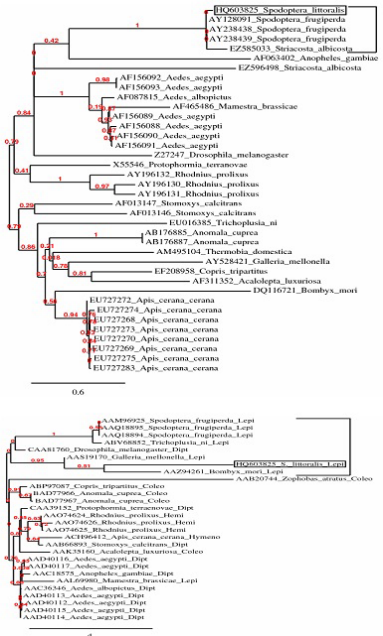
**Phylogenetic analysis of *Spli*Def nucleotide and its deduced amino acid sequences compared to 37 and 27 sequences registered in NCBI**. Phylogenetic trees were generated from 38 and 28 defensin-related sequences by neighbor-joining distance analysis using Phylogeny.fr web service, One Click mode. Full sequence names and accession numbers are included in the tree.

Phylogenetic analyses have been performed on the *Spli*Def nucleotide seuquence and its deduced polypeptide and the results of these analyses are shown in Figure 6. In the case of *Spli*Def nucleotide seuquence, a phylogenetic tree was generated from 38 defensin-related sequences (19 insect species including 7 Lepidoptera, 6 Diptera, 3 Coleoptera, 1 Hymenoptera, 1 Hemiptera and 1 Thysanura) by neighbor-joining distance analysis with maximum sequence difference 1.0 (Figure 6). The topology shows two distinct lineages including 20 (Lepidoptera, Diptera and Hemiptera) and 18 (Lepidoptera, Diptera, Coleoptera, Hymeonoptera and Thysanura) defensin-related sequences, respectively. The maximum nucleotide sequence divergence was exhibited in the first lineage (10 phylogenetic groups). Meanwhile, the defensin sequences appear in the other lineage as less divergent clades (8 phylogenetic groups). The *Spli*Def was clustered with other 4 lepidopteran sequences, *S. frugiperda *defensin (Acc# AAM96925), spodoptericins (Acc# AAQ18895 and Acc# AAQ18894) and *S. albicosta *(Acc# EZ585033), in a monophyletic sister clade (Figure 6). Meanwhile, the other 5 lepidopteran sequences were diverged in different phylogenetic clades (Figure 6). In the case of *Spli*Def deduced amino acid seuquence, a phylogenetic tree was generated from sequence data of 28 published sequences (18 insect species including 6 Lepidoptera, 6 Diptera, 4 Coleoptera, 1 Hymenoptera and 1 Hemiptera) by neighbor-joining distance analysis with maximum sequence difference 1.0 (Figure 6). The topology shows two distinct lineages including 8 (Lepidoptera and Diptera) and 20 (Lepidoptera, Diptera, Coleoptera, Hymenoptera and Hemiptera) defensin peptides, respectively. The maximum divergence of amino acid sequences was exhibited in lineage II (11 phylogenetic groups). However, less divergence was observed in the other lineage (5 phylogenetic groups). The *Spli*Def putative peptide was clustered with other 4 lepidopteran (*S. frugiperda*, *T. ni*, *Galleria mellonella *and *Bombyx mori*) and one dipteran (*D. melanogaster*) defensins in a monophyletic sister clade (Figure 6). Meanwhile, the 5^th ^lepidopteran sequence was grouped in a different phylogenetic group (Figure 6).

### Quantitative protein analysis

Quantitative protein analysis of the crude haemolymph of control and bacterial-challenged *S. littoralis *was determined at 1, 6, 12, 24, 48 and 72 h p.i. (Table 2). Statistical analysis of data revealed that the increase of total protein content in the case of bacterial-challenged insects was significant at all the tested times. *Df, F *and *P *values were illustrated in Table 2. The expected antibacterial peptide concentration in the haemolymph of bacterial-challenged insects was increasing smoothly with the time and an abrupt peak was observed at 48 h p.i. In addition, the total protein concentration of IPTG-induced, non-induced transformed *E. coli *and Ni-affinity purified defensin mature peptide was determined at 1, 2 and 3 h p.i. (Table 2). The protein concentration increased with the time course reaching maximum at 3 h p.i. Statistical analysis of data revealed that the difference of protein content (expressed protein) in the case of IPTG-induced and non-induced cells was significant at all the tested times. *F *and *P *values were illustrated in Table 2. The quantity of protein lost by purification (loss due to purification = induced - non-induced - purified) was 65.8, 167.6 and 60.4 μg at 1, 2 and 3 h p.i., respectively. This loss was statistically significant (*P *= 0.00) in all the tested cases.

**Table 2:** Quantitative protein analysis of the immunized haemolymph of *S. littoralis *and the purified defensin peptide after induction of the recombinant *E. coli *by IPTG.

Protein concentration at different hours post-infection (μg/ml) **Mean ± S.E**.								
	**1 h**	**2 h**	**3 h**	**6 h**	**12 h**	**24 h**	**48 h**	**72 h**

Cont-H	588 ± 6.1	-	-	624.8 ± 6.4	620.2 ± 4.8	651.4 ± 7.5	645.4 ± 5.3	675.6 ± 2.5

Inf-H	687.8 ± 8.9	-	-	908.2 ± 7.2	919.4 ± 4.7	948.8 ± 6.2	2017.6 ± 9.5	1316.4 ± 13.0

Expec. A.B.	99.8	-	-	283.4	299.2	297.4	1372.1	640.8

Induced	909 ± 4.9	1057.2 ± 30.5	1413.2 ± 38.9	-	-	-	-	-

NonInd	702.2 ± 6.0	725.8 ± 6.4	938.8 ± 14.1	-	-	-	-	

Expressed P.	206.8 ± 3.8	331.4 ± 24.4	474.4 ± 36.2					

Purified	141 ± 7.8	163.8 ± 6.4	414 ± 4.0	-	-	-	-	-

Loss	65.8 ± 0.5	167.6 ± 1.3	60.4 ± 0.2					

*df, F, P*	4, 1694.2, 0.0	4, 458.1, 0.0	4, 397.9, 0.0	4, -, 0.0	4, -, 0.0	4, -, 0.0	4, -, 0.0	4, -, 0.0

Cont-H: Untreated crude haemolymph.

Inf-H: Treated haemolymph.

Expec.: Expected antibacterial peptide concentration.

Induced: IPTG induced *E. coli *culture.

NonInd: Non-induced *E. coli *culture.

Expressed P.: (Induced - Non-induced).

Purified: Purified expressed peptide using Ni-affinity column.

Loss: Loss due to purification.

### Antibacterial assay

Table 3 shows a summary of the antimicrobial screening of the immunized haemolymph and the Ni-affinity purified mature *Spli*Def peptide (Figure 2C) based on microbial growth inhibition zone (in mm). Significant activity was found against gram (-) and gram (+) bacteria for both the immunized haemolymph and the purified defensin (Table 3). Notably the antibacterial activities of both immunized haemolymph and purified defensin 48 h p.i. were more than 24 h p.i. for all tested gram (-) bacteria. As for the activity 24 and 48 h p.i. against gram (+) bacteria, no difference was observed in the case of *S. sanguinis *and a slight difference was observed in the case of purified defensin with *S. aureus *bacteria. The antibacterial activity of the immunized haemolymph was comparable to the positive control in the case of *P. vulgaris *and exceeded it against the other tested gram (+) and gram (-) bacteria, 48 h p.i. Meanwhile, the antibacterial activity of the purified defensin was comparable to in the case of *P. vulgaris *and *K. pneumoniae *and more than the positive control against *E. coli*, *S. aureus *and *S. sanguinis *bacteria, 48 h p.i.

**Table 3:** Antibacterial activity of the immunized haemolymph and the purified mature defensin peptide on gram (-) and gram (+) bacteria.

Microorganism	Antibacterial activity			
	
(Gram stain)	Immunized haemolymph		Mature purified peptide	
	
	**24 h p.i**.	**48 h p.i**.	**24 h p.i**.	**48 h p.i**.
*Escherichia coli *(-ve)	++	+++	+	+++

*Proteus vulgaris *(-ve)	+	++	(+)	++

*Klebsiella pneumoniae *(-ve)	++	+++	+	++

*Staphylococcus aureus *(+ve)	+++	+++	++	+++

*Streptococcus sanguinis *(+ve)	+++	+++	+++	+++

(+): Inhibition zone less than 1 mm surrounding the 6 mm paper disk

+: Inhibition less than positive control

++: Inhibition comparable to positive control

+++: Inhibition more than 10 mg penicillin; inhibition zones of references = 13 ± 1 mm diam.

### RT-qPCR

**Figure 7:**
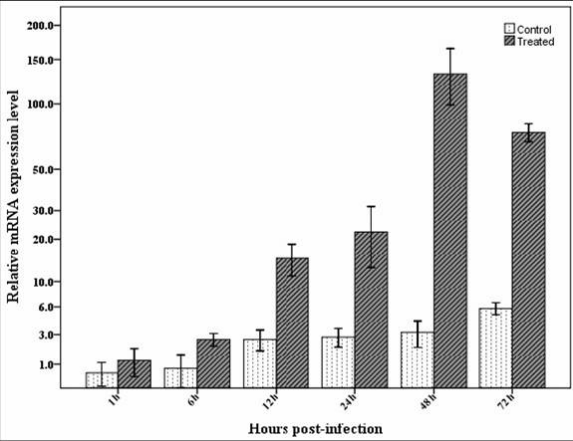
**RT-qPCR of the *Spli*Def from RNA isolated from the haemolymph of control and bacteria-challenged *S. littoralis *larvae at 1, 6, 12, 24, 48 and 72 h p.i. Data were statistically analyzed and compared with control using ANOVA**. Values The bars show the mean ± SE of relative mRNA expression levels.

*Spli*Def transcript profiles from larval haemolymph of the control and the bacterial-challenged *S. littoralis *were compared at 1, 6, 12, 24, 48 and 72 h p.i. using RT-qPCR (Figure 7). The *Spli*Def gene was up-regulated by bacterial-challenge at 1, 6, 12, 24, 48 and 72 h p.i. (2-folds, 3.25-folds, 5.7-folds, 7.9-folds, 41.4-folds and 12.9-folds, respectively). Statistically significant changes were observed between the control and the treated samples at 48 and 72 h p.i. (*df *= 4, *F *= 101.44, *P *= 0.00). Notably, it was more than 41-folds up-regulated at 48 h p.i. No statistically significant change was observed between the control and the treated samples at 1, 6, 12 or 24 h p.i. (*df *= 4, *F *= 101.44, *P *> 0.05).

## Discussion

In the present study, DD-PCR revealed that several common bands were observed in both control and challenged samples (house keeping genes). Very few bands were recorded in control insects and disappeared in challenged ones (genes were turned off). On the other hand, many bands were induced as a result of bacterial-challenge at different time intervals post-infection. DD-PCR technique is considered a powerful genetic screening tool for complicated dynamic tissue processes, particularly when multiple, limited-sized samples are involved, because it allows for simultaneous amplification of multiple arbitrary transcripts [15]. This technique has been developed as a tool to detect and compare altered gene expression in eukaryotic cells [16], to screen mRNAs, and to characterize differentially expressed mRNAs [17-20]. Many publications described the enhancement of the insect immune system and induction of AMPs due to stress and/or bacterial challenge [8-10,12,13,21-24]. Although defensin genes were isolated from six insect orders; Lepidoptera, Diptera, Coleoptera, Hymenoptera, Hemiptera and Thysanura, the lepidopteran defensin-like genes included both the smallest (*G. mellonella *(216 bp), Acc# AAS19170) and the largest (*S. frugiperda *(306 bp), Acc# AAM96925) molecular-sized defensin genes isolated from class Insecta. Thus, the *orf *of *Spli*Def (300 bp) was comparable in size to that of other *Spodoptera *sp. defensin-like genes (303-306 bp).

Reconstruction of the phylogenetic trees of the *Spli*Def nucleotide seuquence and its deduced polypeptide resulted in two different topologies. In spite of constructing two different tree topologies, both trees clustered the *Spli*Def sequence with that of *S. frugiperda *to indicate that they descend from a common ancestor. The grouping of some lepidopteran and dipteran defensins (*e.g*. *M. brassicae *with *A. aegypti *and *S. littoralis *with *D. melanogaster*) in one sister clade indicated that they may be homologous or share some similarity. In addition, the lepidopteran defensin-like sequences were diverged in many sister clades as nucleotides but they were clustered in a monophyletic group as amino acids due to the difference in codon usage in the different insect species.

Although three signal peptide cleavage sites were detected, the most probable site is between positions 23-24. The detected glycosylation and glycation residues may serve for correct folding and stability of the protein. It was shown that the unglycosylated protein degrades quickly. Glycosylation may also play a role in cell-cell adhesion (a mechanism employed by cells of the immune system), as well [25]. In addition, 15 phosphorylation sites were identified. Reversible phosphorylation of proteins (using kinases and phosphatases) is considered an important regulatory mechanism that occurs in both prokaryotic and eukaryotic organisms [26]. It is very important in protein-protein interaction via recognition domains [27,28], (i.e. many proteins and receptors are switched "on" or "off" by phosphorylation and dephosphorylation). It may also result in a conformational changes in the structure of many peptides, causing them to become activated, deactivated or degraded [29].

Spacing pattern of our anionic defensin revealed a possible frameshift mutation of at least six residues within the conserved cysteine motif of *Spli*Def (His^77^, Leu^79^, Lys^81^, Gly^82^, Tyr^83 ^and Gly^86^) in comparison to (His^73^, Leu^75^, Lys^77^, Gly^78^, Tyr^79^, Gly^82^) of BmdefA [14]. Numbers refer to the prepropeptide sequence of a defensin. The first two anionic defensins of *Amblyomma hebraeum *were reported by Lai *et al*. [30]. Similar properties were reported by Wen *et al*. [14] for a novel anionic defensin peptide (PI: 4.12) isolated from *B. mori*. These results were contrary to almost all known defensins, which were described as cysteine-rich cationic AMPs [*e.g*. [11,21].

Although most insect defensins are active against gram (+) bacteria, the purified mature *Spli*Def exhibited activity against both gram (+) and gram (-) bacteria. According to Shai-Matsuzaki-Huang (SMH) model, antibacterial activity was ascribed to the interaction between the positively charged AMPs and the negatively charged microbial membrane components, which include LPSs in gram (-) bacteria and polysaccharides in gram (+) bacteria [31]. However, this model has difficulty explaining the behaviours of anionic defensins like *Spli*Def. For example, the *Amblyomma *defensin-2 contains a net negative charge with a PI value of 4.44, and exerts antimicrobial activity against the gram (-) bacterium *E. coli *and the gram (+) bacterium *S. aureus *[30]. This suggests that anionic defensins might possess some novel antimicrobial mechanisms; although no convincing evidence is available until date. As the knowledge of anionic defensins is still poorly known, it is of interest to investigate the properties of the *Spli*Def.

Sequence motifs similar to the binding sites of TATA, CATT, IL-6-RE, and GATA transcription factors in mammals were found in promoter region of our sequence and almost in all genes that are up-regulated after immune challenge [32]. TATA, CATT, IL-6-RE, and GATA boxes were found adjacent to each other indicating that they work cooperatively in the transcriptional activation of the *Spli*Def gene as previously described in cecropin A_1 _gene in *Drosophila *[33]. Our results also showed that the relative transcription levels of *Spli*Def were up-regulated after bacterial-challenge, indicating the involvement of the *Spli*Def gene in immune responses of the Egyptian cotton leafworm, *S. littoralis*. The expression of *Spli*Def in haemocytes peaks at 48 h p.i. and gradually declines with time. These results were supported by the quantitative protein analysis which revealed the significant peak of increase at 48 h post-infcetion. Kaneko *et al*. [34] reported that the expression of *BmDefensinB *in fat body tissue of *B. mori *peaks at 8 h p.i. and declines with time. Ceraul *et al*. [35] demonstrated that the expression of two defensin isoforms in midgut and fat body tissues of the hard tick *Dermacentor variabilis *peak at 48 h p.i. with *Rickettsia *and gradually decline with time. The expression peak in fat body tissue may be retarded to be at 72 h p.i. in the case of defensin-2. Lopez *et al*. [21] found that the defensin expression pattern in fat body of bacteria-injected *Rhodnius prolixus *tissue is higher than in the intestinal tissue. This pattern may be due to the fact that the expression of defensin gene in deferent body tissues depends on the consequences of infection course of a pathogen (injected pathogen attacks haemocytes and fat body firstly and fed pathogen attacks alimentary canal firstly).

## Conclusions

Our current results provide a typical anionic insect defensin gene (*Spli*Def) with a possible frameshift mutation. *Spli*Def plays an important immune role in *S. littoralis *by cooperating with other AMPs to control bacterial infection and it dominates at 48 h p.i. These findings would be helpful in defensin studies concerning ELISA, PCR and other related molecular and immunological techniques.

## Methods

### Insects and bacterial strains

A laboratory colony of the cotton leafworm, *S. littoralis*, used for our experiments was originally collected from okra field at Giza, Egypt in 1995 and maintained in the insectary of the Department of Entomology, Faculty of Science, Cairo University according to the technique described by El-Defrawi *et al*.[36]. Larvae were reared on a semisynthetic diet described by Levinson and Navon [37] and kept at 25 ± 1°C, 65-70% RH and 14L: 10D photoperiod cycle.

Two gram (+) bacteria, *Staphylococcus aureus *and *Streptococcus sanguinis*, and one gram (-) bacterial strain, *Escherichia coli *(D_31_), were obtained from the Unit for Genetic Engineering and Agricultural Biotechnology, Faculty of Agriculture, Ain Shams University and used for insect immunization. Bacteria were grown in a peptone medium (1%), supplemented with 1% meat extract and 0.5% NaCl, at 37°C in a rotary shaker.

### Bacterial challenge and haemolymph collection

Bacterial challenge was performed by injecting 20 newly moulted fourth instar larvae with 2-5 μl of approximately 1 × 10^6 ^(cells/ml) log phase bacteria dissolved in membrane-filtered saline using a thin-needled microsyringe. Haemolymph was collected 1, 6, 12, 24, 48 and 72 h p.i. at 4°C (500 μl/each), containing few crystals of phenylthiourea to prevent melanization. Haemolymph was pooled by piercing a proleg with a fine, sterile needle. Haemolymph was aliquoted (100 μl each) and stored at -80°C for a weak to be investigated. The same procedures were applied to control group except it was injected with saline without bacteria.

### DD-PCR using primers corresponding to well known defense genes

Total RNA of the insect haemolymph (300-500 μl) was extracted using RNeasy kit according to the manufacturer's instructions (Qiagen, Germany). Residual genomic DNA was removed from RNA using RNase-free DNase (Ambion, Germany). RNA was dissolved in DEPC-treated water, quantified using a BioPhotometer 6131 (Eppendorf) and analyzed on 1.2% denatured agarose gel to ensure its integrity. The 260/280 and 260/230 ratios were examined for protein and solvent contamination.

A total of 100 ng of DNA-free total RNA was converted into cDNA using a mix of random and oligodT20 primers according to the ABgene protocol (ABgene, Germany). Synthesis of the first cDNA strand was performed in a thermal cycler (Eppendorf, Mastercycler 384, Germany) programmed at 42°C for 1 h, 72°C for 10 min and a soak at 4°C. The cDNA was aliquoted and stored at -80 until processed (within a weak).

A total reaction volume of 25 μl containing 2.5 μl PCR buffer, 1.5 mM MgCl_2_, 200 μM dNTPs, 1 U *Taq *DNA polymerase (AmpliTaq, Perkin-Elmer), 2.5 μl of 10 pmol primer (Table 1) and 2.5 μl of each cDNA was cycled in a DNA thermal cycler (Eppendorf, Mastercycler 384, Germany). The amplification program was one cycle at 94°C for 5 min (hot start), followed by 40 cycles at 94°C for 1 min, 40°C for 1 min and 72°C for 1 min. The reaction was then incubated at 72°C for 10 min for final extension. PCR product was visualized on 1.5% agarose gel and photographed using gel documentation system. For DNA contamination assessment, a no-reverse transcription control reaction was performed.

Eight reproducible bacterial-induced bands were eluted, cloned in *PCR-TOPO *vector (Invitrogen, USA) and sequenced using M_13 _universal primer. Sequencing was performed using T^7^Sequencing™ kit (Pharmacia, Biotech, USA) and model 310 automated sequencer (Applied Biosystems, Foster City, CA, USA). Analyses of nucleotide and deduced amino acid sequences were carried out using EditSeq-DNAstar Inc., Expert Sequence Analysis software, Windows 32 Edit Seq 4.00 (1989-1999) and ExPasy database http://expasy.org/tools/dna.html. Blast search for alignment of the obtained sequence with the published ones was done using database of NCBI http://blast.ncbi.nlm.nih.gov/Blast.cgi.

### Full length cDNA cloning and sequence analysis

Based on the sequence and alignment data, specific primers for defensin-related sequences were designed and tried for reverse transcription polymerase chain reaction (RT-PCR). Primers were designed by the rules of highest maximum efficiency and sensitivity. Rules were followed to avoid formation of self and hetero-dimers, hairpins and self-complementarity. RT-PCR reaction was performed as previously described in this section regarding to the optimum annealing temperature (T_a_) for each specific primer set. Positive PCR products were visualized and eluted from the gel using GenClean Kit (Invitrogen Corporation, San Diego, CA, USA) following the manufacturer's instructions. The purified PCR products were cloned into *PCR-TOPO *vector with TOPO TA cloning kit (Invitrogen, USA) following the manufacturer's instructions. Ligation mix was used to transform competent *E. coli *strain TOPO_10 _provided with the cloning kit. White colonies were screened using PCR as described earlier in this section. Five positive clones of *Spli*Def fragment were selected and sequenced (to exclude PCR errors certainly) using the specific forward and reverse primers. To study the characteristics of the promoter region of the *Spli*Def gene, promoter region was amplified from chromosomal DNA (extracted using DNeasy kit following the manufacturer's instructions) using PromF and PromR primers (Table 1). Sequencing and sequence analyses were performed as described early in this section. The core promoter region and the transcription start site of the *Spli*Def gene was predicted using Neural Network Promoter Prediction http://www.fruitfly.org/seq_tools/promoter.html and the minimum promoter score was set at 0.8. In addition to the above mentioned analyses, ExPasy Proteomics Server http://expasy.org/tools was used to calculate physico-chemical parameters of the translated peptide (ProtParam tool). Furthermore, primary and secondary structure analyses, post-translational modifications and topology predictions were investigated using SignalP, NetCGlyc, NetOGlyc, NetGlycate, YinOYang, NetPhos, NetPhosK, Sulfinator, ProP, NetNES, TatP and TMHMM tools. Phylogenetic analyses of the nucleotide sequence and its deduced amino acids were done using Phylogeny.fr web service, One Click mode. Poorly aligned positions and divergent sequences were eliminated manually. Multiple alignment of 58 published defensin-related nucleotide sequences was done before phylogenetic analyses to approximate sequence lengths manually. 100% homologous sequences of the same species with different accession numbers were represented by only one sequence. The cloned DNA fragment was deposited in GenBank under the HQ603825 accession number.

### Expression of the mature defensin peptide

*p*PROEXTM HTa Prokaryotic Expression System kit (Life technologies, USA) was used to clone the purified PCR product corresponding to mature defensin peptide following the manufacturers' instructions. Charged *p*PROEX HTa vector was transformed into the competent *E. coli *strain DH_5_α provided with the kit. Gene expression was induced by IPTG as described by Goh *et al*. [38]. Induced and non-induced samples were analyzed on 12% Tricine SDS-PAGE and the expressed protein was affinity-purified on nickel-nitrilotriacetic acid Superflow resin (Qiagen, Germany) according to the manufacturer's protocol and quantified spectrophotometrically using Bio-Rad protein assay kit (Bio-Rad, USA) following the manufacturer's protocol. Standard curve was constructed by using Bovine gamma globulin (BGG).

### Antibacterial assay

*In vitro *antimicrobial studies of the haemolymph samples as well as the purified mature peptide were carried out by the agar disk diffusion method with minor modifications [39,40]. Five milliliters of 0.6% melted LB agar (52°C) were mixed with 100 *μ*l of viable bacterial suspension (1.6 × 10^9 ^cells/ml), and poured into a 9 cm plastic dish. Five microliters of each haemolymph and protein samples were applied to a 6 mm diameter paper disk and incubated at 37°C. Total protein concentration was quantified spectrophotometrically in both the control and the bacterial-challenged samples using Bio-Rad protein assay kit (Bio-Rad, USA) following the manufacturer's protocol. The difference between the control and the treated samples was considered accumulated AMP in the haemolymph (subtraction method). Standard curve was constructed by using BGG. Haemolymph volumes were corrected for total protein concentration all over the experiment. Penicillin (10 mg/disc; obtained from Sigma) and normal saline solution were used as positive and negative controls, respectively. *E. coli*, *Proteus vulgaris*, *Klebsiella pneumoniae, S. aureus *and *S. sanguinis *were used for testing the antimicrobial activity. Inhibition zone diameters of five replicates were measured after 24-48 h. The degree of growth inhibition was quantitatively evaluated after 16 h by comparison with the growth inhibition resulting from the positive control.

### Quantitative real-time PCR (RT-qPCR)

In order to estimate the comparative transcription rate of defensin, RT-qPCR was used. RT reaction was done as described above. Initially, 1 μl of the RT reaction was diluted and used in a RT-qPCR reaction using defensin primers and untreated control to adjust the sample volume (ensure similar amplification profiles). *18S rRNA *gene was used as reference gene for RNA normalization. Each sample was measured thrice in a 96-well plate (Bio-rad) in a 30 μl reaction volume. Samples were run on a BioRad *i*Cycler machine under the following conditions: 95°C (5 min) for one cycle, and 40 cycles of 95°C (30 sec), 58°C (30 sec) and 72°C (60 sec). The PCR reagents were similar to the regular PCR with the addition of 1 μl of a 1/1000 dilution of Sybr-Green I (Sigma, USA) and 2.5 μl of a 1/1000 dilution of fluorescein to control the background fluorescence. Subsequently, the adjusted sample volumes of cDNAs were used to amplify defensin gene in the haemolymph of control and treated insects at 1, 6, 12, 24, 48 and 72 h p.i. Fluorescent detection was performed at the annealing phase and during subsequent dissociation curve analysis to confirm that a single product had been amplified. The quantification cycles (Cq) were calculated using the iQ5 Optical system software version 2.0. Primer dimers yielded a single sharp peak at the amplicon's melting temperature. No-template and no-reverse transcription reactions were used as negative control. Target amplification efficiency of the reaction was determined from the slope of a plot of Cq versus -log10 concentration of the initial number of target molecules and all assays showed high efficiency of amplification (90-96%) and low intra- and inter- assay variations. All RT-qPCR experiments adhered to the MIQE (Minimum Information for Publication of Quantitative Real-Time PCR Experiments) guidelines [41]. MIQE checklist is attached as additional file 1.

### Statistical analyses

The gene expression levels were quantified relative to the expression of the *18S rRNA *gene using the Gene Expression Macro software (Bio-Rad, Hercules, Calif., USA), employing an optimized comparative Cq (ΔΔCq) value method. One way analysis of variance (ANOVA) and multiple comparison tests (*Scheffé*) were applied to protein concentration, inhibition zone and RT-qPCR results using SPSS (ver17.0) computer software (SPSS for Windows, SPSS Inc.). The scale type of Y-axis was adjusted to the power exponent of 0.25 at a safe mode to improve the quality of chart and to keep the relative difference between the control and the treated samples.

## Conflict of interests

The authors declare that they have no competing interests.

## Authors' contributions

AMS: Designed the overall study, carried out the molecular genetic studies optimization, performed data analysis, interpretation and manipulation, drafted and revised the manuscript, conceived of the study and participated in its coordination. EEH: Participated in the optimization of molecular genetic studies, performed data analysis and interpretation, conceived of the study and participated in its coordination. FHG: Participated in the optimization of molecular genetic studies, performed data analysis and interpretation, conceived of the study and participated in its coordination. All authors have read and approved the final manuscript.

## Supplementary Material

Additional file 1 **MIQE checklist for authors, reviewers and editors**. All essential information (E) must be submitted with the manuscript. Desirable information (D) should be submitted if available. If using primers obtained from RTPrimerDB, information on qPCR target, oligonucleotides, protocols and validation is available from that source. Click here for file
